# Evaluation of an Environmental Transport Medium for *Legionella pneumophila* Recovery

**DOI:** 10.3390/ijerph18168551

**Published:** 2021-08-13

**Authors:** Marianna Martinelli, Enrico Calaresu, Rosario Musumeci, Chiara Giubbi, Federica Perdoni, Sergio Frugoni, Santina Castriciano, Maria Scaturro, Maria Luisa Ricci, Clementina E. Cocuzza

**Affiliations:** 1 Department of Medicine and Surgery, University of Milano-Bicocca, 20900 Monza, Italy; e.calaresu1@gmail.com (E.C.); rosario.musumeci@unimib.it (R.M.); chiara.giubbi@unimib.it (C.G.); federica.perdoni@unimib.it (F.P.); sergio.frugoni@unimib.it (S.F.); clementina.cocuzza@unimib.it (C.E.C.); 2 Copan Italia SpA, 25125 Brescia, Italy; santina.castriciano@copangroup.com; 3 Istituto Superiore di Sanità, 00161 Roma, Italy; maria.scaturro@iss.it (M.S.); marialuisa.ricci@iss.it (M.L.R.)

**Keywords:** *Legionella pneumophila*, culture method, molecular detection, transport media, ISO 11731:2017

## Abstract

The collection and storage of water-related matrices such as biofilm from collection to processing are critical for the detection of *Legionella pneumophila* by cultural and molecular tests. SRK™ is a liquid medium that acts both as an antimicrobial neutralizing agent and a transport medium for bacterial culture enumeration and is useful to maintain the stability of the sample from collection to analysis. The aims of this study were to evaluate *Legionella pneumophila* viability and bacterial nucleic acids’ stability in SRK™ medium over time at different storage conditions. Artificial bacterial inoculates with an approximate concentration of 10^4^, 10^3^ and 10^2^ CFU/mL were made using *Legionella pneumophila* certified reference material suspended in SRK™ medium. Bacteria recovery was analyzed by cultural and molecular methods at time 0, 24 and 48 h at room temperature and at 0, 24, 48 and 72 h at 2–8 °C, respectively. SRK™ medium supported *Legionella pneumophila* culture viability with CFU counts within the expected range. The recovery after 72 h at 2–8 °C was 83–100% and 75–95% after 48 h at room temperature. Real-time PCR appropriately detected *Legionella pneumophila* DNA at each temperature condition, dilution and time point. Results demonstrated a good performance of SRK™ medium for the reliable recovery of environmental *Legionella*.

## 1. Introduction

*Legionella* spp. are aquatic bacteria that are ubiquitously found in nature, in both anthropogenic structures and in environmental waters [1]. The most common pathogenic species is *Legionella pneumophila* (*Lp*) serogroup 1 and is responsible for up to 80% of Legionnaires disease (LD) cases [2]. The exact incidence of LD worldwide is unknown because countries differ greatly in the methods used to ascertain whether someone has the infection and in reporting known cases [3]. In 2019, the incidence of LD in Italy was equal to 52.9 cases per million inhabitants [4].

Environmental sampling for the detection of *Lp* represents an important tool to obtain data of epidemic risk assessment and to provide remedial interventions. Monitoring water systems involves choosing sampling sites, and the number and type of samples to be obtained (water and/or biofilm), as well as the sampling method to be used [5]. Environmental sample storage and transport can be critical for the culture and nucleic acid detection of *Lp*, especially from biofilm samples. Italian guidelines for LD prevention and control suggest the collection of biofilm samples from a specific surface area using a sterile swab subsequently stored in a tube containing 2–5 mL of Ringer, Page or saline solution [6]. These solutions contain different concentration of salts that help to maintain the osmotic balance and allow bacterial enumeration. However, these solutions do not contain components able to inactivate the presence of antibacterial agents that may be present in samples which could interfere with bacterial quantification, such as ammonium, alcohol, and oxidizing and phenolic compounds used for sanitation procedures. SRK™ (Copan Italia SpA, Brescia, Italy) is an alternative liquid medium that acts both as a neutralizing agent of antimicrobial substances and as a transport medium for bacterial culture enumeration. De Filippis and colleagues previously described the use of this medium to evaluate the prevalence of *Legionella* in retirement homes and group homes’ water distribution systems [7]. Another study conducted in Italy evaluated the use of biofilm samples to monitor *Legionella* spp. in hot water systems using 2 mL of Page’s solution for specimen resuspension [8]. Casini and colleagues chose to rinse biofilm samples into 2 mL of water for the investigation of non-culturable *Legionella* spp. in hot water systems [9]. The standardization of biofilm sampling and related analytical practices is still far from being optimized [10], although reference documents [11,12] consider biofilm sampling an essential as part of *Legionella* environmental surveillance [13].

Conventional culture methods represent the gold standard for the detection and enumeration of *Legionella* spp. in water samples, although it can take up to 14 days to obtain a result. Moreover, culture methods are often hampered by the presence of viable but non-culturable (VBNC) *Legionella* and/or by the presence of fast-growing microorganisms that can inhibit the growth of *Legionella*, reducing the sensitivity of the method. Molecular detection, such as quantitative polymerase chain reaction (qPCR), represents an alternative tool for the rapid identification and quantification of *Legionella* in environmental water samples. The main advantages of this technique are the ability to detect *Legionella* contamination at very low levels, the rapid acquisition of results and the easier handling of large sample volumes. However, the interpretation of the results has been largely controversial [14,15,16].

The aim of this study was to evaluate the use of SRK™ medium for the recovery of *Lp* in terms of viability to culture and nucleic acid stability for qPCR detection over time and in different temperature conditions.

## 2. Materials and Methods

### 2.1. Sample Preparation

Artificial bacterial inoculates with an approximate concentration of 10^5^, 10^4^ and 10^3^ CFU (Colony Forming Units)/mL were prepared using dilutions of certified *Lp* lenticules (NCTC 12821 Vitroids™, Sigma-Aldrich, St. Louis, Missouri, USA) in SRK™ medium. Lenticules’ certificate reported CFU values and uncertainties of 583,000 (±54,000) on BCYE agar and 193,000 (±29,000) on GVPC agar, respectively.

**Figure 1:**
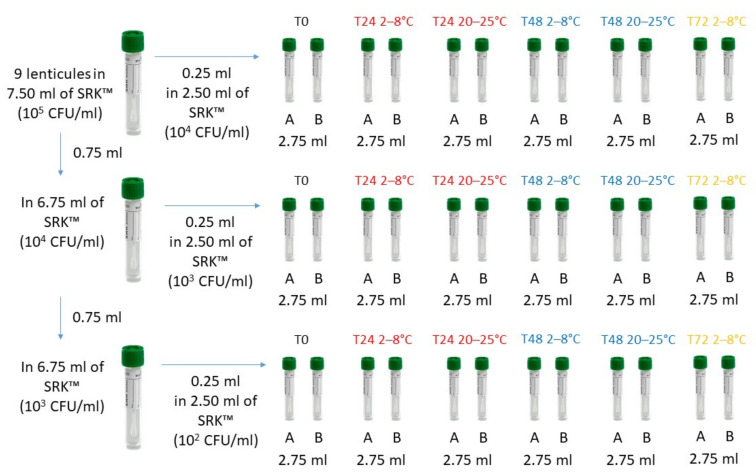
Scheme for the preparation of bacterial suspensions in SRK™ medium with final *Lp* concentrations of approximately 10^4^, 10^3^ and 10^2^ CFU/mL. Bacteria recovery using culture (tube A) and molecular methods (tube B) was evaluated at time 0, 24 and 48 h (T0, T24, T48) following storage at 20–25 °C and at time 0, 24, 48 and 72 (T0, T24, T48, T72) hours at 2–8 °C, respectively.

Nine lenticules were dissolved into 7.5 mL of SRK™ medium to obtain an *Lp* bacterial suspension of 10^5^ CFU/mL (6.9 × 10^5^ and 2.3 × 10^5^ CFU/mL considering BCYE and GVPC agar, respectively). Two further serial dilutions (10^4^ and 10^3^ CFU/mL, respectively) were obtained using 0.75 mL of the bacterial suspension and 6.75 mL of SRK™ medium. These 3 starting bacterial suspensions were further diluted (0.25 mL of each bacterial dilution was added to a further volume of 2.50 mL of SRK™ medium in order to achieve a bacterial suspension of approximately 10^4^, 10^3^ and 10^2^ CFU/mL) in a set of 12 tubes for each concentration, in order to store the bacterial suspensions at different temperatures for recovery at different time points using both culture and molecular methods (tube A and B). The samples preparation procedure is summarized in Figure 1.

To evaluate the ability of SRK™ medium to maintain *Lp* viability and nucleic acid stability, bacterial inoculates were stored in two different temperature conditions: room temperature (20–25 °C) and refrigerated temperature (2–8 °C). Bacterial recovery was evaluated by cultural and molecular methods at time 0, 24 and 48 h (T0, T24, T48) at room temperature and at time 0, 24, 48 and 72 (T0, T24, T48, T72) hours at 2–8 °C, respectively.

### 2.2. Culture Method

Culture media (BCYE and GVPC) inoculate volumes were used as indicated in ISO 11731:2017 [17]. The agar plates were inoculated with 0.1 mL of each dilution and incubated aerobically in presence of 5% CO_2_ at 37 °C for 7 days in a humid atmosphere. Colony counts were performed in triplicate for each bacterial inoculum.

### 2.3. Molecular Methods

Nucleic acid stability in inoculated SRK™ medium was evaluated using both quantitative and qualitative real-time PCR detection assays associated with their nucleic acid extraction kits following manufacturer’s instructions.

Two different commercial kits were used: iQ-Check^®^ Quanti *Legionella* spp. in association with AQUADIEN™ KIT for DNA extraction (Bio-Rad, Hercules, CA, USA); and qualyfast^®^ *Legionella* qPCR detection Kit with qualyfast^®^ DNA Extraction kit I (Bioside, Brescia, Italy). A total of 500 µL of each bacterial dilution was centrifuged for 5 min at 15,000 rpm to obtain a bacterial cell pellet, which was then resuspended using specific buffers, according to the manufacturer’s instruction, respectively. Extracted DNA was eluted in 100 µL of R2 solution of AQUADIEN™ KIT and in 200 µL of qualyfast^®^ *Legionella* buffers (Bioside, Brescia, Italy).

The iQ-Check™ Quanti *Legionella* spp. (Bio-Rad, Hercules, CA, USA) is NF VALIDATION certified (certificate numbers BRD07/15–12/15), and it contains reagents to amplify and quantify *Legionella* spp. Each amplification was performed using 45 µL of reaction mix and 5 µL of samples or controls, according to manufacturer’s instructions. The detection limit of this qPCR method is 5 Genomic Units (GU) per well, corresponding to 80 GU/L. The quantification limit of the method is 608 GU/L.

The qualyfast^®^ *Legionella* product is a kit for detection, discrimination, and quantification of *Legionella* spp. and *L. pneumophila* from water. All the reagents are pre-dosed and lyophilized in the reaction tubes, allowing storage at room temperature. A total of 15 µL of extracted DNA was added to each tube containing lyophilized reagents. A total of 15 µL of DNA free solution was added to positive and negative controls and to calibration scale (external standard). Limits of determination and quantification of this assay are 5 GU and 25 GU per reaction, respectively.

Real-time PCR assays were performed on CFX96 Touch Real-Time PCR Detection System (Bio-Rad, Hercules, CA, USA) following technical sheet. Amplification results were analyzed using CFX Manager™ Software (Bio-Rad, Hercules, CA, USA).

### 2.4. Data Analysis

Data regarding *Lp* growth on agar plates were reported as CFU mean values of plate counts performed in triplicates. Recovery rate was calculated as: (mean CFU at 48 h or 72 h/mean CFU at T0) × 100 for both GVPC and BCYE plates. Expected colonies were evaluated starting from CFU values reported on lenticule certificate and considering the number of lenticules used to prepare each bacterial suspension and volumes used for each dilution (Figure 1). This calculation was performed for both GVPC and BCYE agar plates.

*Legionella* quantification by qPCR method was reported as log_10_ Genome Units (GU)/sample values. *Legionella* GU/sample was obtained following calculation according to the two specific detection kits used.

## 3. Results

### 3.1. Culture Methods

Results obtained from 10^4^ (expected colonies: 2100 and 6300 on GVPC and BCYE, respectively) and 10^3^ (expected colonies: 210 and 630 on GVPC and BCYE, respectively) dilutions showed an uncountable growth on both types of agar plates (not shown). Only the bacterial suspension of approximately 10^2^ CFU/mL allowed for *Lp* bacterial colonies’ enumeration at all tested conditions. In general, the recovery after 72 h at 2–8 °C storage was 83–100% and after 48 h at 20–25 °C was 75–95% based on the expected number of colonies. Results are reported in Table 1.

### 3.2. Molecular Methods

Real-time PCR detection showed a detection signal in keeping with bacterial inocula up to 72 h at 2–8 °C and up to 48 h at room temperature (20–25 °C), indicating bacterial nucleic acid stability in SRK™ medium. Molecular methods allowed the quantification of all three bacterial suspensions in SRK™ medium (10^4^, 10^3^ and 10^2^ CFU/mL) as genomic units (GU).

#### 3.2.1. iQ-Check^®^ Quanti *Legionella* Spp. Real-Time Assay

**Figure 2:**
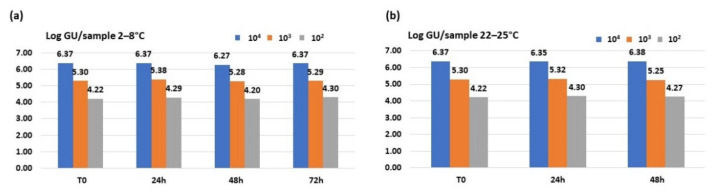
Genomic units/sample detected from 10^4^, 10^3^ and 10^2^ CFU/mL dilutions stored at (**a**) 2–8 °C and (**b**) 20–25 °C using iQ-Check^®^ Quanti *Legionella* spp. Real-Time assay.

Results obtained using iQ-Check^®^ Quanti *Legionella* spp. Real-Time detection assay showed the genomic units remaining stable over time at all three concentrations at both temperatures, as shown in Figure 2. The *Legionella* spp. DNA recovery was almost 99–100% considering all studied dilutions, after 72 h at 2–8 °C storage and after 48 h at 20–25 °C.

#### 3.2.2. Qualyfast^®^ *Legionella* qPCR Detection Kit

**Figure 3:**
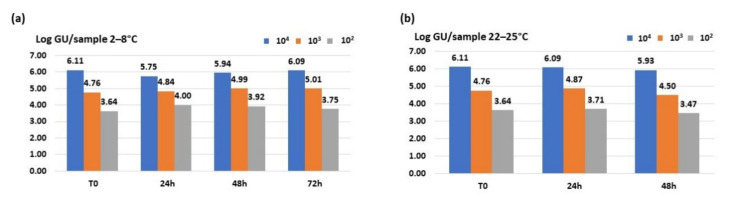
Genomic units/sample detected from 10^4^, 10^3^ and 10^2^ CFU/mL dilutions stored at (**a**) 2–8 °C (**b**) and 20–25 °C using qualyfast^®^ *Legionella* qPCR detection Kit.

Results obtained using qualyfast^®^ *Legionella* qPCR detection kit showed genomic units to be stable over time at all three bacterial concentrations at both temperatures, as shown in Figure 3. An optimal recovery ranging from 95% to 100% was observed using this quantitative real-time PCR kit. Furthermore, this kit also allowed the correct identification of *L. pneumophila* by the use of a second set of primers/probe specific for this bacterial species.

## 4. Discussion

Environmental monitoring represents an important tool to evaluate *Legionella pneumophila* contamination of water systems in order to prevent possible LD outbreaks. Quantification in water samples using culture examination is the gold standard for *Legionella* detection and it is performed according to the International Organization for Standardization [17]. To identify potential *Legionella* presence in water distribution systems, large amounts of water (0.5–1 L) must be collected, and the analysis must be performed within 24 h to ensure that the pathogen remains viable for culture analysis. An alternative way to detect environmental *Legionella* could be the analysis of biofilm samples. It is well known that this pathogen survives as an intracellular parasite of amoebae and protozoa that are found in naturally occurring microbial communities that form biofilms [18,19]. Storage and transport can be critical for *Legionella* culture and nucleic acid detection for both types of specimens.

The aim of this study was to evaluate the bacterial viability by culture and the stability of the nucleic acid detection by PCR of *Legionella pneumophila* in SRK^™^, a liquid medium that acts both as a neutralizing agent for antimicrobial substances and as a transport medium for bacterial culture enumeration.

Regarding culture results, the average number obtained from the bacterial colony counts showed an excellent viability of *Legionella pneumophila* at all tested conditions both by means of growth in the non-selective BCYE medium and in the selective GVPC medium. The colony count averages, resulting from the inoculation of 0.1 mL/plate of the 10^2^ CFU/mL suspension, fell within the CFU/mL count expected for GVPC plates (21 CFU); a slightly higher value was shown in BCYE plates. The inoculation of the same volume from bacterial suspensions at concentrations 10^4^ and 10^3^ CFU/mL showed the growth of uncountable confluent colonies. Cell viability after 72 h at 2–8 °C of storage was found to be between 83 and 100%; after 48 h at room temperature, it was 75–95% as compared to the expected certified lenticule CFU counts. These results indicated that the SRK^™^ conservation medium maintains *Lp* viability over time and at different temperature conditions. Comparing the two different growth media used, a higher number of CFU was observed in the non-selective BCYE medium compared to the selective GVPC medium, in accordance with the lenticule enumeration certificate. This difference in *L. pneumophila* growth is related to the highly selective GVPC composition—a medium enriched with glycine (able to weaken the bacterial wall and favor the action of antibiotics) and with polymyxin B (antibiotic acting against Gram negative bacteria) that could have interfered with the growth of *L. pneumophila* colonies [19].

Even if traditional culture represents the gold standard method to detect *Lp* in water, it can take up to 14 days to obtain a definite result, which is often variable with poor bacterial recovery. The availability of more reliable *Legionella* detection methods could be of great value to rapidly identify contaminated water systems. Molecular biology testing could be a good alternative to the standard culture method due to the high sensitivity and specificity of the results obtained in a shorter time. Several commercial assays that allow the extraction of bacterial DNA and subsequent detection by real-time PCR are currently commercially available. The results of this study demonstrated the high sensitivity and reproducibility of both molecular methods investigated in this study, indicating that the composition of the SRK^™^ medium does not affect the stability and conservation of bacterial nucleic acids and does not interfere with the amplification reagents used in the assays.

Considering the different dilutions made, real-time PCR showed higher values of *Lp* quantification when compared to the culture method on the same samples. Different reasons need to be considered. Real-time PCR cannot distinguish viable from non-viable organisms, although it is able to detect viable but non-culturable (VBNC) *Legionella*. Moreover, the PCR measurement expressed in genomic units per liter cannot be considered equivalent to the unit of measurement for culture methods, expressed in colony-forming units per liter. Despite the clear advantages of molecular methods over culture, the limit of discriminating between live and dead microorganisms remains. Another issue, related to the determination of quantitative *Legionella* GU cutoffs, is to assess the risk of LD. Furthermore, different assays have different sensitivities and different limits of detection; therefore, standardizing molecular method quantification is necessary [20,21,22,23].

Recently, techniques able to inhibit DNA amplification of non-viable cells have been developed to overcome this issue. For example, some studies reported the use of molecules that intercalate into free bacterial DNA or enter membrane-compromised cells, inhibiting qPCR amplification. Propidium monoazide (PMA) is a molecule capable of covalently binding to the non-viable bacterial genome, preventing its amplification [24,25]. However, their use at certain concentrations may have a cytotoxic effect for some bacterial species, such as *Lp* [24]. Another solution, projected to discriminate the viable from the non-viable *Lp* in environmental water samples, is the use of DNase enzymes, able to enter the membranes of dead cells and degrade the nucleic acid of dead bacteria cells. In the future, these innovative approaches could allow the application of molecular biology not only as a screening technique, but also as a confirmation method for the presence of *Legionella* spp. recognized at the level of technical standardization.

## 5. Conclusions

The data obtained in this study showed a good performance of the SRK™ medium, supporting both bacterial viability and nucleic acid stability up to 48 h at room temperature and 72 h at 2–8 °C. SRK™ represents a good collection and transport device for the detection of environmental *Legionella* spp. *iQ-Check^®^* Quanti *Legionella* spp. (Bio-Rad, Hercules, CA, USA) and *qualyfast^®^ Legionella qPCR* (Bioside, Brescia, Italy) also showed excellent recovery of *Lp* in artificially contaminated water samples.

The use of a transport medium such as SRK™ could improve the standardization of both culture and molecular detection of *Lp* from water or water-related matrices, useful for *Legionella* risk assessment evaluation. The water samples’ concentrates, according to ISO 11731:2017, can be tested up to 2 days in the case of epidemic events and with high concentrations of interfering flora. This additional time, by extension, is also applicable to water-related matrices. The good recovery of *Legionella pneumophila* obtained by SRK™ up to 72 h following bacterial inoculation could be taken into consideration for the future updating of ISO 11731:2017.

Further experiments using both *Legionella* not *pneumophila* species and real water and biofilm samples collected from different water systems will be useful in the future in order to confirm the reliability of SRK™ in maintaining *Lp* viability and DNA recovery.

## Figures and Tables

**Table 1:** Mean colony counts (+/−SD) following inoculum of 0.1 mL/plate of 10^2^ CFU/mL dilution.

T (°C)	Agar	T0	24 h	48 h	72 h	Expected Colonies
20–25	GVPC	23 (+/−2)	29 (+/−9)	22 (+/−10)	/	21 (+/−2)
20–25	BCYE	99 (+/−54)	67 (+/−19)	75 (+/−16)	/	63 (+/−9)
2–8	GVPC	23 (+/−2)	43 (+/−22)	48 (+/−9)	28 (+/−11)	21 (+/−2)
2–8	BCYE	99 (+/−54)	63 (+/−36)	72 (+/−30)	82 (+/−51)	63 (+/−9)
